# X-Rays in the Treatment of Malignant Growth

**Published:** 1903-07

**Authors:** Marcus F. Carson

**Affiliations:** Griffin, Ga.


### X–RAYS IN THE TREATMENT OF MALIGNANT GROWTH.

BY MARCUS F. CARSON, M.D.,

GRIFFIN, GA.

The use of the X-rays in the treatment of malignant growths is probably of too recent date to call all of its good results permanent cures.

I have been using this method for nearly two years, and the results in my cases demonstrate its efficiency. They have convinced me that the X-ray is more potent than any other known method in the treatment of certain malignant growths. Cases that were hopeless have been apparently cured and in all that were not completely cured, the progress of the disease has been stayed, and a retrograde metamorphosis established.

In my work I have found that the curative power of the X-ray is not uniform. The growth that has the least tendency to spread is the easiest cured. I believe that until the action of this agent is better understood, until a wider clinical experience teaches us the particular class of cases in which it is most efficient, surgery should precede this method in most primary operable growths.

The action of the X-ray seems to be threefold ; first, stimulant ; second, alterative ; third, destructive. There are varieties in the degree of its action. These degrees are dependent on two things ; first, the nature of the tumor ; second, the quantity of the agent used. If we understood these, X-ray work would be placed on a more scientific basis.

The quantity of this powerful alterative is certainly not the same for all varieties of malignant tissue. Neither do we know the quantity required in any given case. The effect of these different degrees of action on diseased tissue should be very carefully studied.

We know that some growths respond more readily to the degree called a stimulant ; some better to the second or alterative degree, and in some, we can get good results only from the third or destructive degree.

It is the last, or destructive degree, that I have found most dangerous. If we destroy a large mass of malignant tissue, we flood an already depleted system with a virulent toxin, the absorption of which will often produce disastrous results.

It is necessary, therefore, that we study carefully the condition of the patient before applying this agent. With our present knowledge of the physiological action of this powerful agent it is only through a wide clinical experience and observation that we can regulate the quantity in any given case.

Within the last two years I have treated six cases of malignant ulcers, and my results convince me that no other agent or method is so potent in the treatment of inoperable cancer.

My first case was a man, aged fifty-two, a farmer by occupation. Patient presented the following history: About twelve years ago he noticed a small sore on his right eyelid. Month by month this sore increased in size until the whole side of his face was enlarged. Eye ball had sloughed, discharge free, and very foul. Pain constant and very severe. No appetite, and weighed one hundred and eighteen pounds.

On January 8 I gave him his first treatment, and repeated the treatment every third day. After the third application the discharge stopped, pain ceased, and he slept eight hours without waking. As time passed his appetite increased, and in less than two months, he weighed one hundred and forty pounds. In seven months I discharged this patient apparently well, with instruction to return if the sore troubled him again.

CASE 2.—Epithelioma of the cheek of several years' standing. Had been treated by half dozen physicians, with equally as many remedies, but grew steadily worse. After twelve applications, I discharged this patient apparently well.

In these two cases I used the second or alterative degree, which generally leaves a smooth fibrous scar. Six months have elapsed, and there is no evidence of a return in either of these cases.

CASE 3.—Woman, aged thirty-five, with a tumor of left breast about the size of an egg. No glands involved. After thirty-six applications of fifteen minutes each underwent degeneration, and was absorbed.

CASE 4.—A very interesting case of carcinoma of the ear of several years' standing. On this patient I used the third, and most dangerous, destructive degree. The first six applications of twenty minutes each seemed to make it more virulent in its nature and more rapid in its progress. I therefore discontinued treatment. At the expiration of three weeks he returned. I was agreeably surprised to find that area of ulcer had reduced two thirds in size, granulations healthy, no pain and no odor, and patient much improved in general health. I then began treatment, using the stimulating or first degree. The place gradually healed.

Facts are convincing, and the physician who condemns the X-ray treatment of cancer is ignorant of its effects, and his opinion should not influence those of us who are studying the therapeutic effects of this unknown quantity.

---

### TO PREVENT INFECTION.

A practical and helpful series of rules for the sanitary management of contagious and infectious diseases has been prepared by the Palisade Manufacturing Company of Yonkers, and issued in pad form with cover.

It is intended that when called to a contagious case the physician shall sign and hand to the attendant one of these printed sheets of "Precautions to be Observed by Patient, Family and Attendants." This series of rules, couched in plain, every-day English, has been carefully prepared, and the information given is accurate and up-to-date. The delivery of such a signed code of instructions not only impresses the family favorably, but also relieves the physician of all responsibility should any of the necessary precautions be omitted. The advertising of Borolyptol is so arranged that if the physician desires, he can detach all reference to the preparation before handing the directions to the family.

One of these pads (thirty-two sheets) will be mailed to any physician who may apply for same.

